# Structural Simulation of a Bone-Prosthesis System of the Knee Joint

**DOI:** 10.3390/s8095897

**Published:** 2008-09-24

**Authors:** Heiko Andrä, Sebastiano Battiato, Giuseppe Bilotta, Giovanni M. Farinella, Gaetano Impoco, Julia Orlik, Giovanni Russo, Aivars Zemitis

**Affiliations:** 1 Fraunhofer Institut für Techno- und Wirtschaftsmathematik Strömungs- und Materialsimulation, Fraunhofer-Platz 1, D-67663 Kaiserslautern, Germany; 2 Dipartimento di Matematica e Informatica, Università di Catania, viale A. Doria, 6, I-95125 Catania, Italy

**Keywords:** Medical Imaging, diagnostic systems, 3D segmentation, FEM, stress simulation

## Abstract

In surgical knee replacement, the damaged knee joint is replaced with artificial *prostheses*. An accurate clinical evaluation must be carried out before applying knee prosthe-ses to ensure optimal outcome from surgical operations and to reduce the probability of having long-term problems. Useful information can be inferred from estimates of the stress acting onto the bone-prosthesis system of the knee joint. This information can be exploited to tailor the prosthesis to the patient's anatomy. We present a compound system for pre-operative surgical planning based on structural simulation of the bone-prosthesis system, exploiting patient-specific data.

## 1. Introduction and Motivation

The knee joint can be severely damaged due to a variety of causes, such as arthritis or knee injury. This can cause pain and inability to walk. In some cases, replacing parts of the joint is thus the appropriate course of action. A total knee replacement is a surgical procedure whereby the damaged knee joint is replaced with artificial shells (*prostheses*), tied to the bone using a special cement. An accurate clinical evaluation must be carried out before applying knee prostheses to ensure optimal outcome from surgical operations.

Most patients suffer from long-term problems, such as *loosening*. This occurs because either the cement crumbles or the bone melts away from the cement. In some cases, loosening can be painful and require reoperation. The results of a second operation are not as good as the first, and the risks of complications are higher. The accurate choice of materials can improve prothesis durability. However, loosening can be mainly avoided (or at least postponed) by tailoring the implanted prosthesis to the patient's anatomical peculiarities. Studying the bone-prosthesis system and its evolution, estimating the forces that will be acting on the prosthesis being implanted, can help to improve the lifespan of the implanted material and tailor the prothesis to the patient's anatomy.

Navigation systems for positioning of orthopaedic implants in individual bones are used in more and more hospitals, and can be considered as the state of the art in orthopaedic surgery. These systems take into account the patient-specific bone geometry and calculate the mechanical axes of the bones. However, only these axes are considered when positioning the implant, excluding important information about the inner bone structure (e.g., the interface between spongy and cortical bone parts, stiffness of these parts, age, gender etc. of the patient, local degradation and reduction of the bone density, presence of sclerotic (very hard) islands inside the bone). The surgeon is aware of all these individual structural pathologies only after opening the bone, so that she has to choose an appropriate prosthesis during the operation.

In the leading European teeth and jaw surgery, this decision is planned on the basis of advanced structural analysis of the implant stability, where CT or cone beam-CT images of the patients provide the interior structure of the bone. Since the CT-image techniques are rapidly developing and CT-devices are commonly available in hospitals, the natural vision in the orthopaedic prosthetic field, and particularly for knee-replacement surgery, is to empower the existing navigation systems with mechanical structural simulation capabilities. This paper is a contribution to the proof of this concept.

Our aim is to design a simulation system that combines methods and algorithms which are simple and robust, so that most of the phases in the modelling and simulation chain can be automated. This methods should also be fast enough, so that the simulation process can be run online during the operation.

The only previous attempt in such direction we know about is *MedEdit* [1], a system that helps surgeons in operation planning and post-operative outcome evaluation. Although this system is a useful tool for analysis and visualisation, it has a number of drawbacks that reduce its usability in the clinical practice. Namely,

- The final model is represented as a triangle mesh, but the interior density of the bone must be known for stress estimation
- The algorithm to extract and classify bone structures from medical data works on a slice-by-slice basis, rather than using the dataset as a whole; this reduces the chance of detecting long structures spanning over different slices; moreover, it can detect only cortical structures
- The segmentation requires too much human intervention, since the user must set a number of seed points to initialise the algorithm and clean the resulting images from incorrectly assigned pixels
- No error control/estimation is provided by the various sub-units of the tool

In this paper, we present a system that is similar in scope to this work, but aims at overcoming its limitations. We employ robust, automated algorithms for CT image classification and finite element mesh generation. All simulation steps are integrated in a tool with a user friendly graphical interface which is easy to manage by medical doctors and surgeons. This project is the joint work of a multidisciplinary team including a prosthesis production company, LIMA spa [2], and the Radiology Department of the Vittorio Emanuele Hospital of Catania, Italy.

## 2. System Overview

The system described in this paper simulates the structural properties of the bone-prosthesis system of the knee joint. This study is motivated by the need of more accurate pre-operative planning procedures for knee-replacement surgical intervention. The aim is a full, highly automated diagnostic system for pre-operative guidance. Among other important issues, this system can help surgeons to select the prosthesis that best fits patient's anatomy among a wide range of sizes, or to design a custom-made one. To our knowledge, this is the first compound system trying to answer to all the technical issues involved in such an ambitious task.

**Figure 1.**
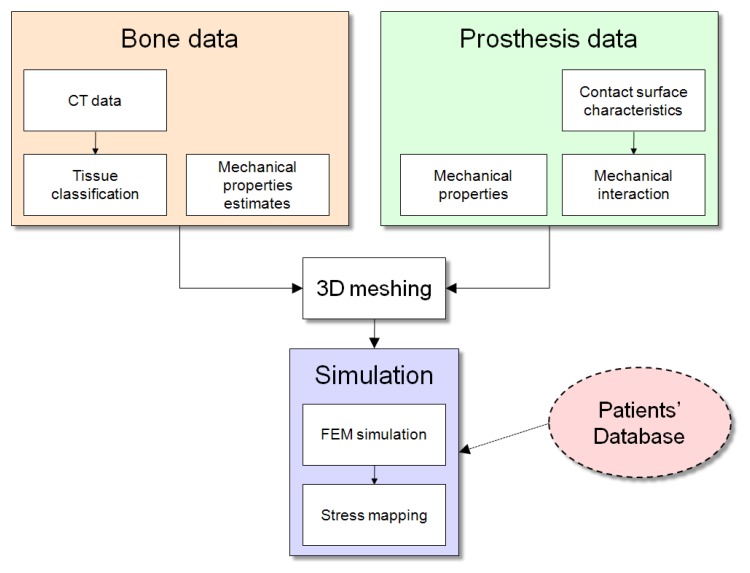
Scheme of our system for pre-operative planning of knee replacement surgery.

Figure 1 shows a schematic diagram of our system for pre-operative planning of knee replacement surgery. Various pieces of data are collected from different sources and processed. The first block is related to bone features: computed tomography (CT) scans and mechanical properties. CT volume elements (*voxels*) are classified into four different tissue classes (see Section 3.2.), in order to extract the geometry of *cortical* (external) and *trabecular* (internal) parts of the bones. Then, mechanical properties of the bone are estimated and mapped onto the CT scan (Section 3.3.). The second block encloses mechanical parameters and surface characteristics of the prosthesis, given by the manufacturer and plugged into the system (Section 4.). A detailed mathematical description of the bone-prosthesis contact is developed (Section 5.). Mechanical data of the tibia and femur, and of the prosthesis are used together with geometry for 3D meshing (Section 6.1.). The last block is related to simulation. 3D meshes and loading conditions are used for FEM simulation (Section 6.1.). A method to deal with uncertainties in the measurements has also been studied (Section 6.3.). The simulated stress results are mapped onto the geometry of the bones for analysis and visualisation. Typical mechanical parameters for the specific clinical case can also be taken from statistical studies about similar cases in the literature, using patients' info such as age, weight, and sex.

In the following sections we discuss each of these issues.

## 3. Bone Modelling

We model two aspects of bone structures for stress simulation: geometry and mechanical parameters of the bone. Geometry is extracted from CT scans of the patients' knee joint using by means of an automatic classification algorithm. A statistical generative model is employed together with a *Maximum A-posteriori Probability* (MAP) classification rule [3]. The probability distributions used for classification are automatically learned from manually-annotated training scans. CT scans are used for two main reasons. First, acquiring CT data for planning is common clinical practice before knee replacement intervention. Second, CT scans give sufficiently accurate data for knee replacement surgery, as pointed out in [4].

From a mechanical viewpoint, the bone is modelled as a three dimensional viscoelastic material. The two regions composing tibia and femur, cortical and trabecular, posses highly different properties that must be taken into account for an accurate stress simulation. These properties are measured by means of a load test on small bone samples cut out from the bone at different positions. Then, they are mapped to the geometry for stress simulation.

### 3.1. CT Data

The first step in creating a bone model out of medical volumetric data is to extract tissue information from this data [5]. In this section, we address the problem of classifying CT data in order to tag cortical and trabecular bone structures.

CT devices output volumetric scans arranged in stacks of 2D images, called slices*. CT scans can be geometrically characterised by three factors: *spatial resolution* along slices (i.e., pixel size), *inter-slice distance*, and *slice thickness*. Slice thickness is the portion of physical volume related to a pixel, i.e., the value of a pixel is influenced by the tissues lying a given distance apart along the normal to the slice plane (axial view). This feature is responsible for the *partial volume (PV) effect*, i.e., the value of a pixel is a combination of the contributions of each tissue enclosed in the volume related to the pixel.

Typical values for spatial resolution and inter-slice distance are, respectively, 0.5 x 0.5 mm, and ∼ 1 mm. Thus, CT datasets can be thought as 3D grids with elongated voxels.† Due to this anisotropy, extending common segmentation algorithms to 3D is not straightforward, since most of them are designed for isotropic data. Slice thickness is usually either equal to inter-slice distance, or twice as big. That is, either the voxels touch or they overlap.

Overlapping slices can increase the PV effect leading to less neat differences between neighbouring voxels belonging to different tissues. On the other hand, this modality offers a number of advantages. First, due to the strong coherence between neighbouring slices, information about a pixel can be largely inferred by the corresponding pixels in the neighbouring slices. Second, if some knowledge is available for a slice, it can be safely propagated to neighbouring slices since they share a portion of their volumes. Third, each structure in the data influences both the voxels sharing an overlapping volume. This data replication can help balancing the PV effect. Finally, this modality is common clinical practice in most radiology departments.

**Figure 2.**
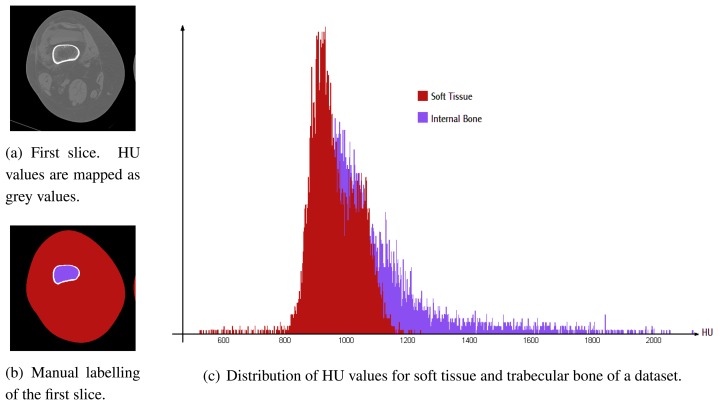
A CT scan of a 80-years-old patient, affected by osteoporosis and arthrosis. A manual labelling is also shown together with the relative distribution of *Hounsfield Units* (HU) values of two tissue classes: *soft tissue and trabecular bone*.

Apart from the dataset geometry, another factor plays a major role for data segmentation: the meaning and range of data values. The content of a voxel is a scalar value, usually in the range [−1000, 3000], expressed in *Hounsfield Units* (HU). HU values are roughly proportional to the density of the material in the sampled volume associated to the voxel. For example, water has a HU of about zero, and cortical bone HU values are usually greater than 1000. This is of great help for segmentation of tissues on the basis of their density. However, the range of values for different tissues often overlap. This is especially true at articulations and for soft tissues and trabecular bone in aged patients, where osteoporosis reduces the density of bones (see Figure 2). Thus, data values cannot be uniquely associated with specific tissues i.e., the data cannot be partitioned using voxel intensity alone. This makes the classification task more challenging and rules out simple thresholding techniques. Moreover, defining a similarity function between neighbouring pixels is hard, since the same tissues often have uneven distributions of intensity values in different areas (e.g., the cortical tissue of the femur has lower HU values in areas close to articulations). Hence, region-growing or edge detection algorithms are unable to effectively cope with this data. Finally, automatically separating adjoining bones, as needed by stress simulation, is a hard task due to PV effects generated by the limited spatial resolution of the CT in the axial direction.

Nine knee datasets were imaged at the Radiology Department of the Vittorio Emanuele Hospital of Catania. The data were captured by means of a Multidetector CT Scanning (MDCT) device in spiral mode, using the acquisition parameters reported in Table 1. The age of the patients ranged from 70 to 80 years and all of them suffer from osteoporosis and arthrosis. This choice is motivated by the fact that this is one of the most difficult cases (due both to age and to severe osteoporosis and arthrosis) and the most widespread in clinical cases of knee replacement surgery. The acquired datasets were manually labelled by expert radiologists of the Vittorio Emanuele Hospital. 75% of the labelled datasets were used for learning, and the remaining were employed for testing.

A large variety of segmentation methods have been developed for medical image processing. Nonetheless, ad-hoc solutions are often preferred to properly detect complex structures [6], such as vessels, organs, or skeletal structures. Most of them do not provide uncertainty estimates about segmentation results. This is a serious drawback for mechanical simulations, since they require a reliable error measure. Finally, and most important, segmentation algorithms group pixels into regions but do not label regions with a semantic meaning. Hence, they lack a real classification mechanism.

Active contours [7] and level sets [8] have been effectively used for image segmentation. They provide smooth, closed contours and can be coupled with statistical methods. However, active contours must deal with special cases due to contour sampling, while level sets are too computationally cumbersome for practical use in clinical practice.

### 3.2. Classification and Volume Segmentation

Since our classification model is intended to be used for surgical pre-operative planning, one of our main objectives is computing, and possibly bounding, the classification uncertainty. This is a good property because it is intuitive and is easily related to background knowledge of medical experts. Basically, one would accept the output of classification if the algorithm is *p*-percent sure about the result. Hence, one can choose to classify only the pixels whose probability of belonging to a certain class is above a user-defined threshold. Uncertain classifications can be regarded as instances of the partial volume effect. We choose this possibility, and let uncertainties go through meshing (Section 6.1.) up to the stage where uncertainties are modelled for stress simulation (Section 6.3.).

**Figure 3.**
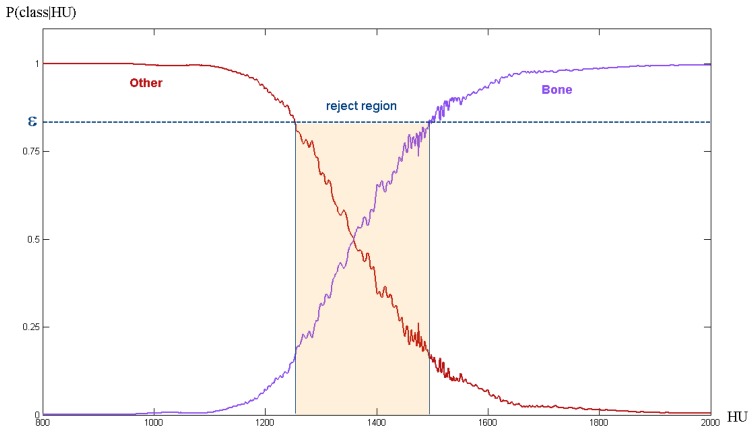
Rejection rule. Probability distributions for two classes, conditioned by the HU value (our simplest feature). The threshold *ε* is used for rejection of low-probability classifications (see text). The shadowed region depicts the rejection interval.

A statistically-founded approach is used in [9] to infer the percentage of different tissues within each voxel due to the *partial volume* effect. Each voxel is modelled as a mixture of contributions from different tissues. Tissue mixture are searched to maximise the posterior probabilities of observing the corresponding data. However, rather than labelling real data to compute HU distributions, they assume that the HU values of each tissue class are normally distributed. Our collected data show that this assumption does not hold for the knee region (Figure 3(c)). A learning procedure is clearly needed to build a reliable classification system.

Our approach [10] employs a simple generative model to classify voxels in a CT dataset into four classes: void volume, soft tissue, trabecular bone, and cortical bone. The likelihood functions involved in the computation of posterior probabilities are modelled by Gaussian Mixture Models (GMM), and are learned using the Expectation-Maximisation (EM) algorithm. The data we used for supervised learning were manually labelled by the radiologists of the Vittorio Emanuele Hospital in Catania. Our algorithm shares some similarities with Lu et al.'s [9] method. While they model voxels as mixtures of contributions from different tissues, we use a single label for each pixel. This may seem a simplification, since our model cannot capture explicitly partial volume effects of the data, as they do. There are two reasons for this choice. First, there is no straightforward manual labelling procedure for supervised learning models. Basically, one can ask radiologists to give a single classification for each pixel (crisp classification), not to guess the percentage of a pixel occupied by a certain tissue (fuzzy classification). Second, there is a loss in classification granularity since partial volume effects are not explicitly modelled by the classification mechanism. However, reduced granularity is countervailed by the greater discriminative power of learned statistics.

Suppose we want to partition our data into *N* different classes. We denote with *c_k_* the *k*-th class. Let us define a number of feature functions *F*_1_(.), …, *F_M_*(.) on the pixel domain. We use a simple generative approach [3] to learn the model for the posterior probability *P* (*c_k_* ∣*F*_1_(*z*), …, *F_M_*(*z*))) for each pixel z. We model the likelihoods *P* (*F*_1_(*z*), …, *F_M_*(*z*)∣*c_k_*) using GMMs (one for each class). Manually-annotated training datasets are used to learn the likelihoods, using the EM algorithm [3]. We assume that the priors *P*(*c_k_*) are uniformly distributed. One might argue that these probabilities are different for each class and that we can compute them from our training sets by counting the number of pixels in each class. However, too many uncontrolled elements can affect the percentage of bone pixels over the total volume, such as biological factors (e.g., age, sex, osteoporosis), and machine setup parameters (e.g., percentage of patient tissues contained into the imaged volume). Hence, equiprobability of the priors *P*(*c_k_*) is a reasonable choice. Assuming equiprobable priors, by the Bayes' theorem we get

(1) P(ck∣F1(z),…,FM(z)))=P(F1(z),…,FM(z)∣ck)∑Pk(F1(z),…,FM(z)∣ck)

where the evidence can be expressed in terms of likelihood functions alone.

The posterior probabilities *p̂*_k_(*z*) = *P* (*c_k_*∣*F*_1_(*z*), …, *F_M_*(*z*))) are used to classify unseen data. A MAP rule is used to get crisp classifications. For each pixel *z* the most probable labelling is

(2) $$C(z)=\arg\max\left[\hat{p}k(z)\right]$$

with associated probability *p̂_C_* (*z*) = max [*p̂_k_* (*z*)]. Being conservative, a strict rejection rule is employed to retain only highly probable classifications (low uncertainty). Thus, we accept this classification if *p̂_C_* ≥ *ε* where *ε* is a user-defined threshold which bounds the classification uncertainty. If we restrict to two classes, *c*_1_ and *c*_2_, and to a single feature *F_1_*(*z*) for the sake of visualisation, our simple rejection option can be depicted using the diagram in Figure 3. Pixels classified as cortical bone with high uncertainty are used for FEM simulation as well (see Section 6.), accounting for this classification uncertainty.

As pointed out before, the HU values of pixels do not suffice to get a robust classification. Pixel values can be affected by noise which rules out simple pixel-wise partitioning methods. Moreover, the distributions HU values of different classes can largely overlap (Figure 2) especially for aged patients or for patients affected by osteoporosis or arthrosis. For this reason, we employ a more robust pixel analysis by looking at the neighbourhood, which can give useful information to estimate the probability of pixels to belong to a certain class, given their surrounding context. Hence, we employ a set of features at different scales to capture the variability of HU values in the surrounding context of a pixel.

**Figure 4.**
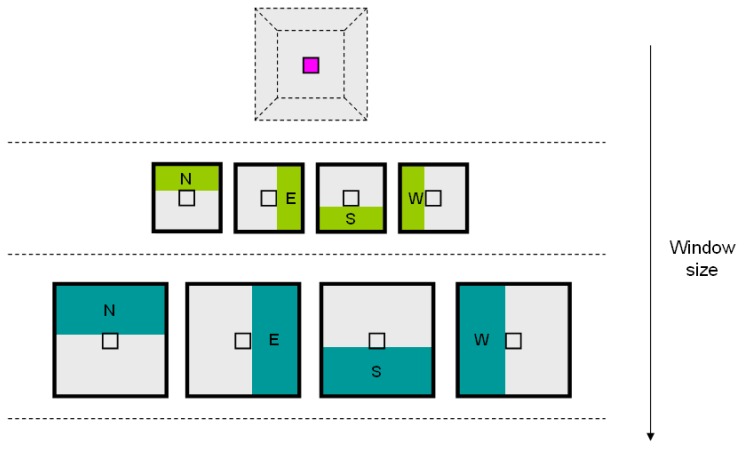
Multiscale feature descriptors employed.

For the sake of exposition, we discuss our multiscale approach for a slice. For each scale s, we employ a *s* × *s* window centred around the interest pixel. We define *υ_s_* = (*F_s,N_*(.), *F_s,E_*(.), *F_s,S_*(.), *F_s,W_*(.)) as the mean of the HU values for four regions in the surrounding window: North (N), East (E), South (S), and West (W) (see Figure 4). In our implementation, we use the HU value of the interest pixel together with the feature vectors *υ_s_*. We choose these features to selectively evaluate the surrounding context of a pixel in four directions. We do not use circular features since they would bring less useful information, being independent from orientation. We also use simple mean instead of more complex distance weighting since distance is accounted for using multiple scales. Due to the dimension of feature windows, especially in larger scales, a special treatment should be reserved to border pixels. In our implementation, we do not bother about image borders since in our application interest pixels always lie in the central area of CT scans.

This model is easily extended to handle volumetric data, provided that the anisotropy of voxels is taken into account. Namely, than taking the same number of samples along the slice plane and across slices would favour the direction orthogonal to the slices. Hence, the neighbourhood of the interest voxel is chosen to be approximately cubic.

The learned model is used to assign to each pixel the probability to belong to a certain class. The MAP rule discussed above can be used to get the final crisp classification. Alternatively, the user can enforce a threshold on these probabilities to bound the classification uncertainty. A volumetric model is generated by labelling cortical and trabecular bone voxels with low uncertainty. Voxel with high uncertainties are regarded as partial volume voxels.

**Figure 6.**
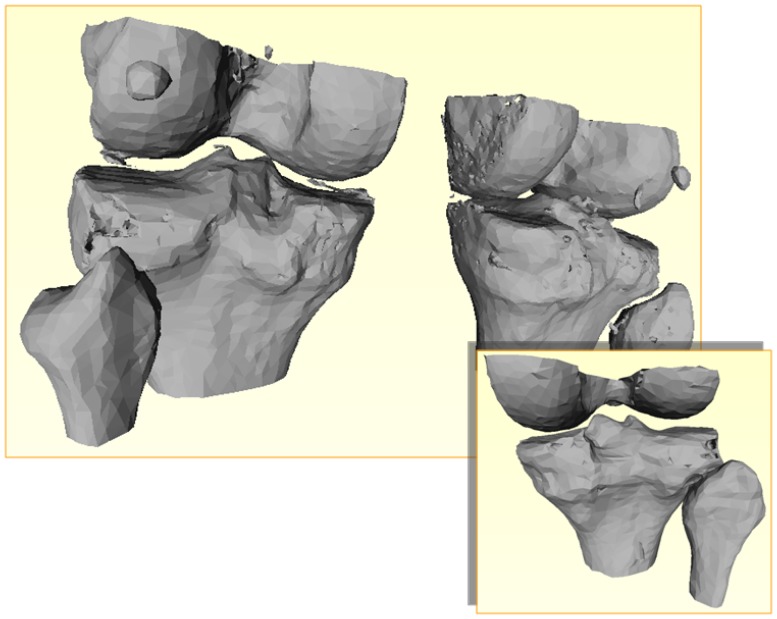
Surface model showing of the cortical tissue of a knee joint. Notice that the the bones of the articulation are neatly separated.

Figure 6(b) shows an example of crisp classification, obtained using our approach with a MAP rule. In this example, the cortical part is broken due to misclassification. This prevents the simulation algorithm to work at all. Hence, a mechanism must be introduced to force the cortical bone to be closed at all times. The method we use is straightforward and works in two steps. First, a thin layer of cortical bone is artificially drawn to separate soft tissue from internal (trabecular) bone (see Figure 6(c)). Second, the user is asked to select the cortical part of the bone of interest by clicking into a single slice. The outline of this selection is deemed as the outer layer of the bone and, most important, is perfectly closed. This thin outline is then expanded to a width specified by the user as a fraction of the diameter of the bone section (Figure 6(d)). Notice that this is anatomically consistent, since the width of cortical bone is proportional to its size. This information is then propagated to the whole dataset, without any human intervention. The voxels of neighbouring slices are marked if they are classified as cortical tissue and lie close to the final cortical bone of the current slice. The cortical area to which the marked voxels belong is selected and the outline is used to repeat the process.

As shown in Figure 6, this algorithm produces the desirable result of keeping bones separated. Hence, it is little sensitive to the partial volume effect.

### 3.3. Bone Mechanical Parameters

**Figure 8.**
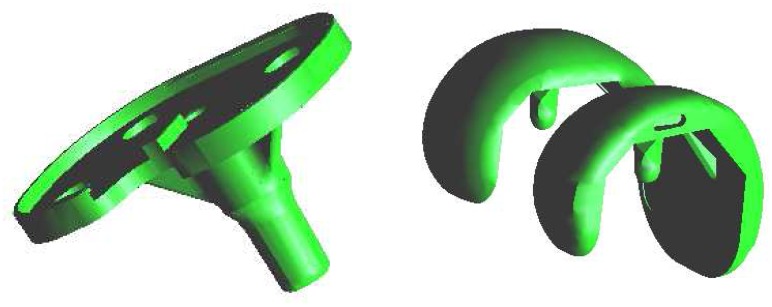
Metal tibial tray (on the left) and metal femoral component (on the right)

The bone is modelled as a three dimensional viscoelastic material. The cortical region is much stiffer than the trabecular part and has a higher density, therefore it carries most of the load. To a first approximation, each region is considered homogeneous and isotropic, with the viscoelastic parameters determined by means of relaxation tests [11, 12]. Small cylindrical samples of the bone are cut out, both from the cortical and trabecular regions. Then, the samples undergo a load test with an ad hoc micro-compression machine (Figure 8(a)). This machine applies a sudden (compressive) strain to the sample and keeps it constant during the tests. A force transductor measures the reacting compressive force as a function of time (relaxation test).

The bone is modelled as a five parameter viscoelastic material, as illustrated in Figure 8(b). The parameters are determined by fitting the measured response using the analytical solution of the relaxation predicted by the model (Figure 8(c)).

Non homogeneity of the bone might be also taken into account. Several cylindrical samples should be cut from the bone both in longitudinal and radial directions with respect to the main axis of the bone, and their mechanical properties should be measured as above. Doing this way, one obtains the parameters in a set of specific points in space. The properties for all points can be obtained by interpolation.

## 4. Prosthesis Modelling

In the case of total knee replacement, the damaged cartilage and bone are removed and replaced by a man-made surface of metal and plastic. The main parts of a knee prosthesis are: plastic patellar component, metal femoral component, plastic tibial spacer, and metal tibial tray. In our approach we investigate only the two main parts of the prosthesis: metal femoral component and metal tibial tray (see Figure ??).

Our aim is to simulate interactions between the tibial tray and a tibial bone and between the femoral component and a femoral bone. It is assumed that both metal parts are linear elastic materials. The prosthesis is not simulated separately from the bone. Therefore, an important step is positioning of the prosthesis in a cut bone and the grid generation for the bone-prosthesis system. This is described more in details in the next sections. A scheme for boundary conditions applied for bone-prosthesis system is depicted in Figure 13

## 5. Mathematical Description and Multi-Scale Modelling of the Bone-Prosthesis Contact

Individual characteristics of patient's bone structure are accounted for in biomechanical models by employing specific material laws and their parameters. The contact zone between bone and prosthesis is of great importance. The local mechanical strain produces a biological stimulus, which leads to bone building [13–15] and to in-growing of the new bone tissue into the pores of the rough coating of the prosthesis. Different microscopic roughness and mechanical properties of the coating imply different effective contact parameters, such as contact stiffness, *k_n_* = *σ_n_*/*u_n_*, and friction coefficient, *µ* = |*σ_τ_*|/*σ_n_*, on the interface between bone and prosthesis.

In this section we discuss a highly accurate multi-scale homogenisation approach developed for the analysis of the contact zone, which allows to calculate the effective macroscopic contact stiffness and friction coefficient on the basis of the microstructure of the coating as well as the elastic properties for the coating, prosthesis and bone materials. The precalculated effective contact parameters are used in the finite element simulation of the mechanical contact problem for the prosthesis-bone system.

**Figure 10.**
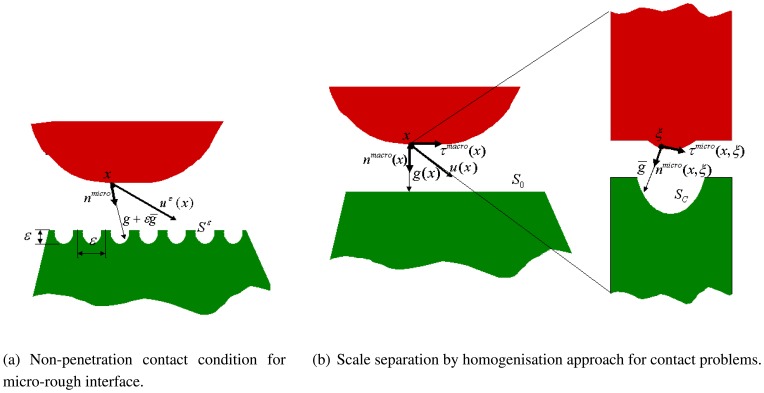
Local and two-scale non-penetration conditions.

Let **u**^*ε*^ be the interface jump in the displacement vector, *ε* be the small parameter, denoting the ratio between the coatings roughness and the macro-size of the bone-prosthesis system; **n***^micro^* denotes the unit outward normal vector of the bone and *g* + *εg̅* is the initial interface gap (see Figure 10). Then, the local non-penetration condition for each coating point *x* ∈ *S^ε^* (see [16]) is

(3) $${\text{u}}^{\varepsilon }(\text{x})\cdot {\text{n}}^{\text{micro}}\left(\text{x},\frac{\text{x}}{\varepsilon }\right)\leq \text{g}(\text{x})+\varepsilon \bar{\text{g}}\left(\frac{\text{x}}{\varepsilon }\right)\cdot$$

The following proposition about non-standard anisotropic macroscopic non-penetration condition was proved in [16] by using a two-scale asymptotic homogenisation technique:

**Proposition.** For the local non-penetration contact condition (3), the anisotropic macroscopic non-penetration condition will be given in the form

(4) $$\{\begin{array}{c} k_{nn}{u_{n}}^{\text{macro}}(x)+k_{n\tau }{u_{\tau }}^{\text{macro}}(x)\leq k_{gn}g(x)\;\text{for a}.\text{a}.x\in S_{0}\\ k_{\tau \tau }{u_{\tau }}^{\text{macro}}(x)+k_{n\tau }{u_{n}}^{\text{macro}}(x)\leq k_{g\tau }g(x),\;\text{for a}.\text{a}.x\in S_{0}, \end{array}$$

where the effective contact parameters *k_nn_*, *k_nτ_* and *k_ττ_* can be found for each known surface micro-profile from the following formulas

(5) $$\begin{array}{l} k_{\alpha \beta }=\frac{1}{\left|T\right|}\int_{S_{C}^{\text{real}}(\sigma^{\text{macro}})}(\alpha^{\text{micro}}(x,\xi )\cdot \alpha^{\text{macro}}(x))(\alpha^{\text{micro}}(x,\xi )\cdot \beta^{\text{macro}}(x))ds_{\xi },\\ k_{ga}=\frac{1}{\left|T\right|}\int_{S_{C}^{\text{real}}(\sigma^{\text{macro}})}(\alpha^{\text{micro}}(x,\xi )\cdot {\text{n}}^{\text{macro}}(x))(\xi )ds_{\xi },\alpha ,\beta =n,\tau . \end{array}$$

The diagonal and tangential macro-stiffnesses, *k_nτ_* and *k_ττ_*, will imply some tangential drag forces, which can be interpreted as friction forces. The drag forces caused by *k_nτ_*, *k_ττ_* can be represented in the form of the Coulomb's friction with the homogenised friction coefficients $\mu =\frac{|k_{n\tau |}}{k_{nn}}+\frac{k_{\tau \tau }}{k_{nn}}\left|\frac{u_{\tau }}{u_{n}}\right|$. This macroscopic contact condition will be used for numerical computations of the macro-problem on. The main result here is that even *starting with the frictionless contact micro-problem with a rough interface, we end up with a macro-problem containing friction*.

**Figure 11.**
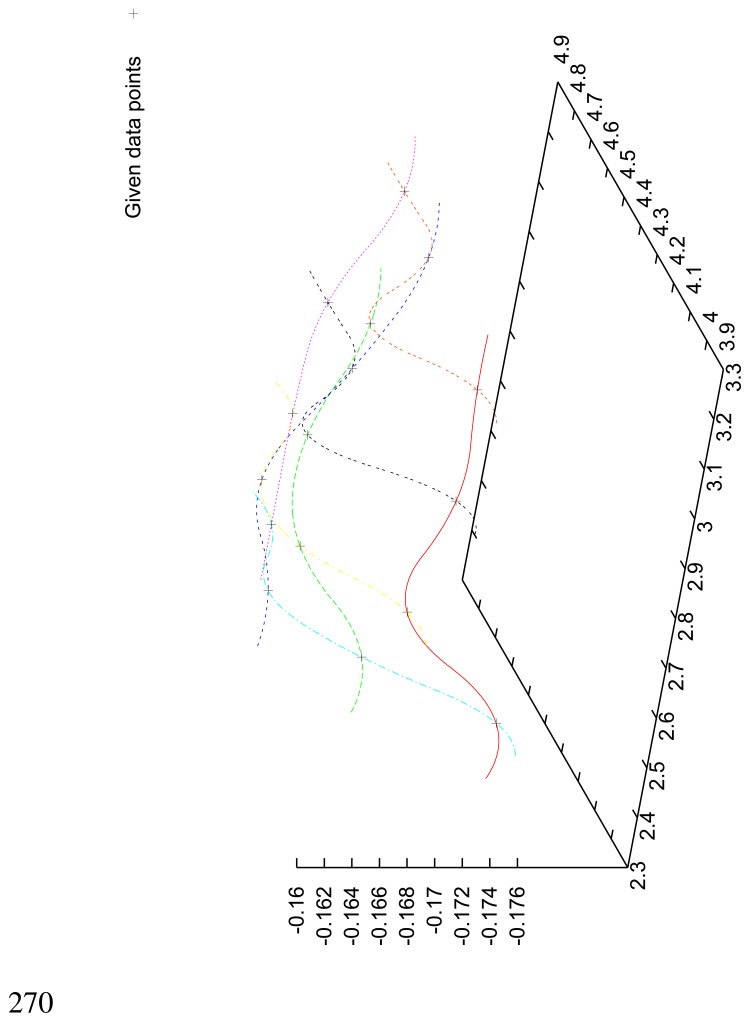
Fragment of the coatings surface interpolated by parabolic splines using given data points.

In Table 2, the normal contact stiffness, *k_nn_*, and the friction coefficient, *µ*, for four coating layers with different porosity and roughness are calculated for the case of the full micro-contact between the bone and the prosthesis. The coatings' surfaces were given as volumetric voxel grid, containing key-points. We interpolated the surfaces by orthogonal parabolic splines (see Figure 11) to obtain coordinates of the micro-normal vector in each point of the micro-contact surface by the standard formula $n(x,y)=\frac{\left(-z_{x}(x,y),-z_{y}(x,y),1\right)}{\sqrt{1+z_{x}^{2}(x,y)+z_{y}^{2}(x,y)}}$. It can be seen that for given materials the magnitude of the friction coefficient can differ in several orders. The friction coefficient strongly depends on the roughness and the porosity of the coating layer.

According to Equation 5, the contact coefficients depend on the actual micro-contact area and are independent of the stress-state only in the case of the full microscopic contact. In general, the micro- as well as the macro-contact surface is unknown, depends on the contact stresses, and can be found after solving the micro- as well as the macro-contact problem.

In many phenomenological contact formulas (see, e.g., [17]), contact conditions are assumed in the from the stress displacement relations:

$$\sigma_{n}\left(h\right)=c_{n}\;h^{b_{n}},\;\sigma_{\tau }\left(h\right)=c_{\tau }\;h^{b_{\tau }}$$

where *c_α_*, *b_α_*, *α* = *n*, *τ*, are material constants and the average penetration depth *h* is introduced as a displacement of the mean line of the rough surface. According to [18], *c_n_*, *c_τ_* are proportional to $\frac{1}{\frac{1-v_{\text{Prosth}}^{2}}{E_{\text{Prosth}}}+\frac{1-v_{\text{coat}}^{2}}{E_{\text{coat}}}+\frac{1-v_{\text{Bone}}^{2}}{E_{\text{Bone}}}}$, where *E_coat_*, *ν_coat_* are effective Young's modulus and Poisson's ratio of the coating calculated from the simple analytic Hashin composite sphere model (Christensen [19]) on the basis of its porosity and elastic properties of the coating alloy, *E_alloy_*, *ν_alloy_*. Furthermore, according to our terminology, *h* =< *k_nn_u_n_* + *k_nτ_u_τ_* ‒ *g_n_* > and < *g_n_* >= *Rt*/2. Here < · > denotes the averaging in the cross-section orthogonal to the macro-normal i.e. $<\cdot >:=\frac{1}{\left|S_{0}\right|}\int_{S_{0}}\cdot d\hat{x}$. We obtain then the following macroscopic contact law:

(6) $$\begin{array}{l} \sigma_{n}^{\text{mac}}\approx -\frac{1}{\frac{1-v_{\text{Prosth}}^{2}}{E_{\text{Prosth}}}+\frac{1-{\hat{\nu }}_{\text{coat}}^{2}}{{\hat{E}}_{\text{coat}}}+\frac{1-v_{\text{Bone}}^{2}}{E_{\text{Bone}}}}(k_{nn}<u_{n}>+k_{n\tau }<u_{\tau }>-Rt/2),\\ \sigma_{\tau }^{\text{mac}}\approx -\frac{1}{\frac{1-v_{\text{Prosth}}^{2}}{\mu_{\text{Prosth}}}+\frac{1-{\hat{\nu }}_{\text{coat}}^{2}}{{\hat{\mu }}_{\text{coat}}}+\frac{1-v_{\text{Bone}}^{2}}{\mu_{\text{Bone}}}}(k_{\tau \tau }<u_{\tau }>+k_{n\tau }<u_{n}>). \end{array}$$

For the three coatings considered in Table 2, we performed many micro-contact simulations under different macroscopic contact stresses and displacements and approximated these experiments by the following formulas:

(7) $$k_{\alpha \beta }\left(u\right)\approx a_{a\beta }\text{(}<u_{\tau }>/<u_{n}>)\cdot <u_{\alpha }{>}^{b_{\alpha }-1},\;\alpha ,\beta =n,\tau ,\;b_{\alpha }\in \left[0.3;0.6\right].$$

**Figure 12.**
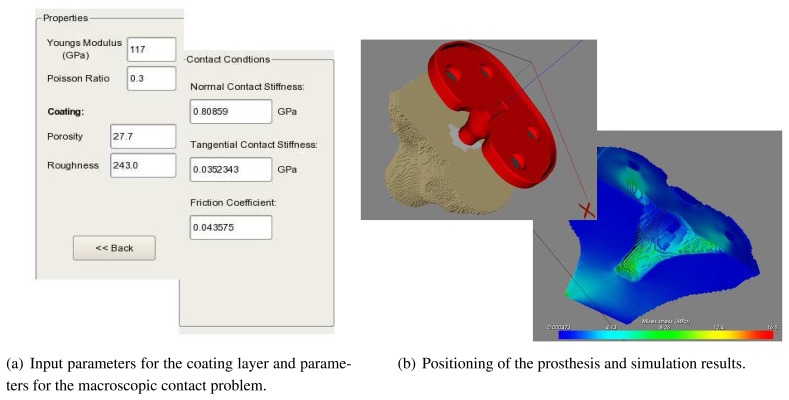
Simulation sequence: set the properties of the prosthesis, obtain contact conditions, position the prosthesis, and solve the macroscopic contact problem.

The calculated parameters for *k_n_* are presented in Table 2. The proposed method for estimation of macroscopic contact conditions is implemented in the software KneeMech [20]. The steps for performing simulations can be seen in Figure 12. The macroscopic contact stiffness *k_n_* and friction coefficient µ are calculated by using the mechanical parameters for the prosthesis and the coating as well as porosity and roughness of the coating. After that, the prosthesis is manually positioned using a GUI. Then, the full mathematical macroscopic contact problem for the bone-prosthesis system can be constructed. It consists of equilibrium equations (8) with constitutive elastic relations (9) for the bone and prosthesis, contact (3), (11) and boundary (12) conditions:

(8) $$\text{div}\sigma (x)=\text{f}(x),\;x\in \Omega_{\text{Bone}}\cup \Omega_{\text{Prosth}}$$

(9) $$\sigma (x)=\frac{E_{K}}{2(1+v_{K})(1-2vk)}\mathrm{divu}u(x)+\frac{E_{K}}{2\left(1{+}_{\nu \kappa }\right)}\nabla \text{u}(x),\;x\in \Omega_{K},\;K\in \left\{\text{Bone},\text{Prosth}\right\}$$

(10) $$k_{nn}{\left[u\right]}_{n}(x)+k_{n\tau }{\left[u\right]}_{\tau }(x)\leq k_{gn}g(x)\text{for}\;\text{a}.\text{a}.\;x\in S_{0}$$

(11) $${\left[\text{u}\right]}_{\tau }=0,\;\text{as long as}\;|\sigma_{\tau }|\leq \mu |\sigma_{n}|\;x\in S_{0,}$$

(12) $$\begin{array}{l} {\left[\text{u}\right]}_{\tau }=-\lambda \sigma_{\tau },\;\text{if}\;|\sigma_{\tau }|=\mu |\sigma_{n}|\;x\in S_{0,}\\ \sigma \cdot \text{n}(x)=\text{t}(x)\;x\in \Gamma_{N,}\\ \text{u}(x)={\text{g}}_{0}(x),\;x\in \Gamma_{u}\cdot \end{array}$$

**Figure 13.**
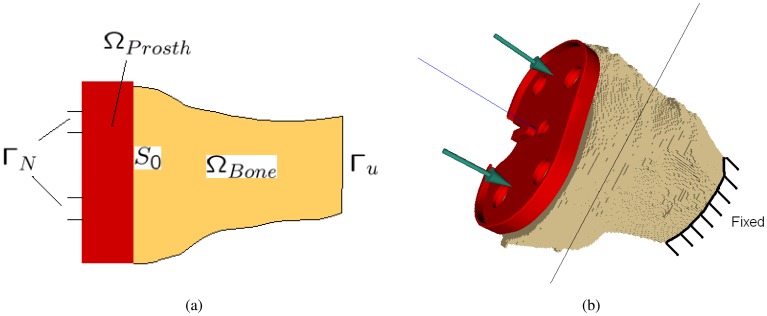
Resulting bone-prosthesis system and scheme of boundary conditions. Arrows represents directions of the loading forces. The bottom part of the bone is fixed.

Here, *σ_n_*(*x*) = (*σ*(*x*) ·**n**(*x*)) ·**n**(*x*) is the normal stress, *σ_τ_*(*x*) = *σ*(*x*) *·***n**(*x*) − *σ_n_***n**(*x*) denotes the tangential stress vector, ${\left[u\right]}_{n}\left(x\right):=(\text{u}\left(x\right)|S_{0}^{\text{Prosth}}-\text{u}(x)|S_{0}^{\text{Bone}})\cdot n(x)$, is the jump in the normal displacement, [**u**]_*τ*_ = [**u**] − [*u*]_*n*_**n**(*x*) is the vector of jumps in the tangential displacements, go and t are components of given vectors of the boundary displacements and traction (see Figure 13 for g_0_ = 0).

Finally, the macroscopic contact problem (8)-(12) will be solved by finite element method.

## 6. Simulation

**Figure 14.**
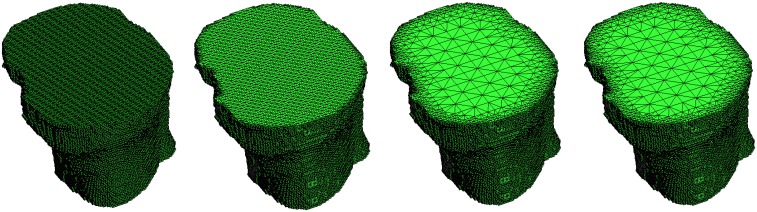
Tetrahedral mesh of the tibia with four levels of coarsening corresponding to the values given in Table 3.

The bone-prosthesis contact problem (8)-(12) describes the behaviour at the interface between the bone and the prosthesis (Figure 14(a)). The simulation of the whole bone-prosthesis system uses a finite-element discretisation. It is thus necessary to convert the voxel representation of the bone into 3D mesh with tetrahedral elements. A coarsening algorithm is employed to reduce the number of finite elements. A penalty method [21-25] is used to solve the resulting linear system. We also provide a sensitivity analysis method to handle uncertainties in the estimation of the mechanical parameters (see Section 3.3.).

### 6.1. Finite Element Discretisation

Let us denote Ω = Ω_*Bone*_ ∪ Ω_*Prosth*_, 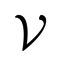 = { υ_i_ ∈ *H*^1^ (Ω), *i* = 1,…, *N* | υ_i_(*x*) = *g*_0*i*_(*x*), *x* ∈ Γ_*u*_}. The elasticity problem without contact can be rewritten in a weak formulation as follows: find *u_i_* ∈ 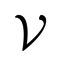 such that for all *υ_i_* ∈ 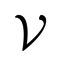

(13) $$\int_{\Omega }a_{\text{ijkl}}\frac{\partial u_{k}(x)}{\partial x_{l}}\frac{\partial (\upsilon_{i}(x)-u_{i}(x))}{\partial x_{j}}dx+\geq \int_{\Omega }f_{i}(x)(\upsilon_{i}(x)-u_{i}(x)-u_{i}(x))dx+\int_{\Gamma_{N}}t_{i}(x)(\upsilon_{i}(x)-u_{i}(x))ds.$$

Since the tensor of elastic coefficients is symmetric, problem (13) is equivalent to the minimisation of the functional:

(14) $$\begin{array}{ll} I(\text{v}) & =\frac{1}{2}\int_{\Omega }a_{\text{ijkl}}\frac{\partial \upsilon_{k}(x)}{\partial x_{l}}\frac{\partial \upsilon_{i}(x)}{\partial x_{j}}dx\\ & -\int_{\Omega }f_{i}(x)\upsilon_{i}(x)dx-\int_{\Gamma_{N}}t_{i}(x)\upsilon_{i}(x)ds \end{array}$$

on the set of *υ_i_* ∈ 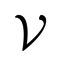.

For convenience we will introduce the following notations:

(15) $$a(\text{v},\text{v})=\int_{\Omega }a_{\text{ijkl}}\frac{\partial \upsilon_{k}(x)}{\partial x_{\iota }}\frac{\partial \upsilon_{i}(x)}{\partial x_{j}}dx,$$

(16) $$f(\text{v})=\int_{\Omega }f_{i}(x)\upsilon_{i}(x)dx+\int_{\Gamma_{N}}t_{i}(x)\upsilon_{i}(x)ds,$$

The domain Ω is represented by a domain Ω_*h*_ consisting of tetrahedral elements. Each component of displacements over each element is approximated by linear polynomials. On the basis of these tetrahedra it is possible to generate a system of piecewise linear global basis functions {Φ_ξ_}. Using this basis we can span a space $V_{h}=\left\{\upsilon_{i}\in C\left({\bar{\Omega }}_{h}\right),i=1,\ldots ,N|\upsilon_{i}\left(x\right)=g_{0i}\left(x\right),x\in \Gamma_{u}^{h}\right\},$ where $\Gamma_{u}^{h}$ is an approximation of the boundary Γ*_u_* by the triangles correspondingly to Ω_*h*_.

Now, if (**v**_*h*_)_*i*_ is an approximation of the i-th component of the displacement field defined over Ω̅_*h*_ then

(17) $$\begin{array}{cc} {\left({\text{v}}_{h}\right)}_{i}(x)=\sum_{\xi =1}^{N_{p}}\upsilon_{i}^{\xi }\Phi_{\xi }(x)≕\upsilon_{i}^{\xi }\Phi_{\xi }(x), & x\in {\bar{\Omega }}_{h}, \end{array}$$

where $\upsilon_{i}^{\xi }≔{\left({\text{v}}_{h}\right)}_{i}\left(x_{\xi }\right)$ and *N_p_* is the total number of grid points. The term (15) in (14) can be approximated as follows:

(18) $$a_{h}\left({\text{v}}_{h},{\text{v}}_{h}\right)=A_{\alpha \beta }^{ij}\upsilon_{j}^{\beta }\upsilon_{i}^{\alpha },$$

where

(19) $$A_{\alpha \beta }^{ik}=\int_{\Omega_{h}}a_{\text{ijkl}}\frac{\partial \Phi_{\beta }}{\partial x_{\iota }}\frac{\partial \Phi_{\alpha }}{\partial x_{j}}dx.$$

An analogous approximation can be applied to the item (16):

(20) $$f_{h}\left({\text{v}}_{h}\right)=f_{\alpha }^{i}\upsilon_{i}^{\alpha },$$

where

(21) $$f_{\alpha }^{i}=\int_{\Omega_{h}}f_{i}\Phi_{\alpha }dx+\int_{\Gamma_{N}^{h}}t_{i}\Phi_{\alpha }ds,$$

and $\Gamma_{N}^{h}$ is an approximation of the boundary Γ_*N*_ by a finite element mesh.

The resulting finite element approximation of the problem can be defined as follows: find (**u**_*h*_)_*i*_ ∈ 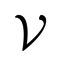
_*h*_, *i* = 1, ..,*N* which minimises the functional

(22) $$I_{h}^{\delta }({\text{v}}_{h})=\frac{1}{2}a_{h}({\text{v}}_{h},{\text{v}}_{h})-f_{h}({\text{v}}_{h})$$

The problem (22) is equivalent to solving the linear equation system. If contact conditions (6) are taken into account then the problems becomes non-linear and the corresponding problem must be solved iteratively. The simplest way for taking the contact conditions into account is by using of penalty method and a successive iterative method [24]. In this case, the contract stress is calculated on the basis of known displacements $u_{h}^{m}$ in the *m*-th iteration and the $u_{h}^{m+1}$ can be found by minimising a similar functional:

(23) $$\begin{array}{rr} I_{h}^{\delta }({\text{v}}_{h}) & =\frac{1}{2}a_{h}({\text{v}}_{h},{\text{v}}_{h})-f_{h}({\text{v}}_{h})+\frac{1}{\delta n}{\int_{\Gamma Ch}\left[k_{nn}{\left[{\text{u}}_{h}^{m}\right]}_{n}\left(x\right)+k_{n\tau }{\left[{\text{u}}_{h}^{m}\right]}_{\tau }(x)-k_{gn}g(x)\right]}_{+}{\left[{\text{v}}_{h}\right]}_{n}ds\\ & +\frac{1}{\delta n}{\int_{\Gamma Ch}\left[k_{\tau \tau }{\left[{\text{u}}_{h}^{m}\right]}_{\tau }\left(x\right)+k_{n\tau }{\left[{\text{u}}_{h}^{m}\right]}_{n}(x)-k_{g\tau }g(x)\right]}_{+}{\left[{\text{v}}_{h}\right]}_{\tau }ds, \end{array}$$

where *δ_n_*, *δ_τ_* > 0 are small penalty parameters, chosen with respect to (6) as follows:

$$\begin{array}{c} \delta_{n}=\frac{1-v_{\text{Prosth}}^{2}}{E_{\text{Prosth}}}+\frac{1-{\hat{v}}_{\text{coat}}^{2}}{{\hat{E}}_{\text{coat}}}+\frac{1-v_{\text{Bone}}^{2}}{E_{\text{Bone}}},\\ \delta_{\tau }=\frac{1-v_{\text{Prosth}}^{2}}{\mu_{\text{Prosth}}}+\frac{1-{\hat{v}}_{\text{coat}}^{2}}{{\hat{\mu }}_{\text{coat}}}+\frac{1-v_{\text{Bone}}}{\mu_{\text{Bone}}}, \end{array}$$

Γ*_Ch_* is the discretised contact surface and [.]_+_ ≔ *max* {0,.}. It is important to remark that all nodal points in Γ*_Ch_* are interface points. This means that in these points the displacements can have different values in different materials. The corresponding linear system is solved using a preconditioned conjugate gradient solver [26].

### 6.2. Hierarchical mesh coarsening

The numerical method for solving the elasticity problem for the bone-prosthesis system uses a tetra-hedral mesh. Therefore, a conversion step from the voxel data to a tetrahedral mesh is required. The simplest way is to start with a straightforward decomposition of a single voxel into five tetrahedrons. This approach leads to a very large number of finite elements. The number of tetrahedra is five times the number of voxels used. For real time applications it is necessary to reduce the finite element system. Therefore, we developed an ad-hoc hierarchical mesh coarsening algorithm.

The idea is to apply local coarsening operations to neighbouring voxels being part of the same material. Namely, if a small cubic neighbourhood of eight voxels lies entirely in the same structure the voxels are combined to form a larger unit. This procedure is run recursively with the restriction that neighbouring voxels differ at most in one level of coarsening. The advantage of this constraint is that the resulting mesh shows smooth transitions from small elements at boundaries and interfaces to larger element in the inner regions.

In order to make compatible mesh elements at different coarsening levels, we follow the approach proposed by [27] and [28]. The computational time for mesh generation is reduced since all possible tetrahedra which occur can be calculated in advance and stored in a look-up table.

Depending on the geometry, mesh coarsening can dramatically reduce the number of nodes and elements, especially for large connected components. The maximum number of hierarchical levels of the mesh can be controlled in order to avoid the creation of large elements causing a larger discretization error. Reduction factors and computational times are shown in Table 3 for a triangulated tibia. Simplifications up to a factor of six can be obtained. Some coarsening steps of the mesh are shown in Figure 14.

### 6.3. Dealing with Uncertainties

Determining the mechanical parameters for the bone of the patients is an error-prone process. As such, they can only be determined up to a given precision. Modern mathematical tools for self-verified computing allow us to solve the finite element problem considering the uncertain nature of the mechanical parameters. Given the data as ranges of possible values, self-verified computing returns the range of possible values for the results, or at least an outer bound (overestimation) of the actual physically possible range.

Classical self-verified methods, however, such as those based on the Interval Analysis (IA) developed in the ‘60s by Moore [29], are not suitable for application to finite element methods (FEM) when the number of nodes and elements is very large. Naive methods will incur in extremely large bound overes-timation, sometimes even failing to give meaningful results. More specialised methods, such as the one developed by Muhanna [30], which gives much better bounds, become computationally prohibitive as the problem becomes large, since they cannot exploit the sparseness of the stiffness matrix.

Therefore, we developed a radically different approach based on Affine Arithmetic (AA), a self-verified mathematical model developed by Stolfi and de Figueiredo [31]. This allows us to obtain very quickly a first-order approximation of the result range. If further precision is needed, Muhanna's IFEM method can be used to calculate the reminder. The final range is guaranteed to be more compact than the range obtained using Muhanna's method alone.

Another significant advantage of our approach is that the uncertain problem is decomposed in a number of deterministic, classical FEM problems with the same left-hand side. All but one of these sub-problems are independent, and therefore the problem is highly parallelisable. The number of problems is equal to *N_c_* + 1, where *N_c_* is the number of uncertain mechanical parameters.

The method is tuned to handle uncertainties in the mechanical parameters on which the stiffness matrix depends linearly. For example, the Young's modulus *E*, the Lame constants λ and *µ*, and so on. For the viscoelastic problem with mechanical parameters *E_0_*, *E_i_*, *η_i_*(*i* = 1,2) (see also section 3.3.) solved with timestep Δ*t*, uncertainty modelling should consider expressions such as

$$E_{i}\frac{1-\exp\left(-\frac{\Delta t}{\eta i}\right)}{\frac{\Delta t}{\eta i}}$$

as a single uncertain parameter, to preserve linearity.

For simplicity of exposition we will assume in what follows that only one mechanical parameter per element is uncertain, and precisely the Young's modulus. For the finite element *ε*^*(i)*^ we can write its Young's modulus *E*^(i)^ in the form:

$$E^{\left(i\right)}=E_{0}^{\left(i\right)}+E_{j}^{\left(i\right)}\varepsilon_{j}$$

where *ε_j_* is a *noise symbol*, an unknown such that |*ε_j_*| < 1. $E_{0}^{\left(i\right)}$ is the *central value* of the Young's modulus, while $E_{j}^{\left(i\right)}$ is the radius of its uncertainty.

Since the local stiffness matrix depends linearly on *E^(i)^*, we can write it in the form

(24) $$K^{\left(i\right)}=K_{0}^{\left(i\right)}+K_{j}^{\left(i\right)}\varepsilon_{j}$$

where $K_{0}^{\left(i\right)}$ comes from the central value of *E^(i)^* and $K_{j}^{\left(i\right)}$ comes from the Young's modulus uncertainty $E_{j}^{\left(i\right)}$.

The noise symbol index *j* is different from the finite element's index *i* because it is possible for different elements to use the same noise symbols; this happens, when their uncertainties is assumed to be correlated. For example, under the assumption that the bone is homogeneous, we will have a single noise index shared by all the trabecular elements, and a single (different) one for the cortical elements, giving us a problem with two uncertainties.

More complex situations are also possible, up to the extreme case of a noise index (i.e. a distinct uncertainty) for each element: this situation however is not physically sensible, as nearby elements are likely to have highly correlated mechanical parameters.

Regardless of the number of uncertainties *N_c_*, however, the global stiffness matrix is obtained combining local matrices in the form of Equation 24, and it will therefore have the form:

$$K=K_{0}+\sum_{i=1}^{N_{c}}K_{i}\varepsilon_{i}$$

where *K*_0_ is the same matrix that would be obtained in the deterministic problem (ignoring uncertainties), and the *K_i_* are *N_c_* matrices obtained from the uncertainties.

Since boundary conditions don't usually depend on the uncertainties, the left-hand side matrix of our finite element problem takes the form *M* = *M_0_* + Σ *M_i_ε_i_* where

$$\begin{array}{cc} M_{0}= & \left[\begin{array}{cc} K & B^{T}\\ B & 0 \end{array}\right],\\ M_{i}= & \left[\begin{array}{cc} K_{i} & 0\\ 0 & 0 \end{array}\right] \end{array}$$

The form assumed by our problem when uncertainties are take into account is therefore

(25) $$(M_{0}+\sum M_{i}\varepsilon_{i})u=f$$

where *u* is the vector of the unknowns (displacements and lagrangian parameters) and *f* is the given right-hand side.

Since the dependency of *u* from *M* is nonlinear in the *ε_i_*, we can write *u* as the sum of a linear part *u_a_* and a linearisation of the nonlinear part *u_l_*. The linear part will be in the form

$$u_{a}=u_{0}+\sum u_{i}\varepsilon_{i}$$

so we can write explicitly

$$(M_{0}+\sum M_{i}\varepsilon_{i})(u_{0}+\sum u_{i}\varepsilon_{i}+u_{\iota })=f$$

and by developing the product, we have

$$M_{0}u_{0}+\sum (M_{i}u_{0}+M_{0}u_{i})\varepsilon_{i}+\sum M_{i}u_{j}\varepsilon_{i}\varepsilon_{j}+Mu_{\iota })=f.$$

which must be satisfied for every choice of *ε_i_*, *i* = 1,…, *N_c_* in [0,1]. This can only be satisfied (polynomial identity rule) if the coefficients of *ε_i_* on each side of the equation are equal, and therefore it must be

$$\begin{array}{cc} M_{0}u_{0} & =\;f,\\ M_{i}u_{0} & +\;M_{0}u_{i}=0\;\forall i=1,\varepsilon ,N_{c},\\ \sum M_{i}u_{j}\varepsilon_{i}\varepsilon_{j} & +\;Mu_{\iota }=0 \end{array}$$

which we rewrite as

(26) $$M_{0}u_{0}=f,$$

(27) $$M_{0}u_{i}=-M_{i}u_{0}\;\forall i=1,\ldots ,N_{c},$$

(28) $$Mu_{\iota }=-\sum M_{i}u_{j}\varepsilon_{i}\varepsilon_{j.}$$

The first two equations show that the terms in the linear part of the solution can be calculated by solving for *N_c_* + 1 sharp problems, all with the same coefficient matrix (*M_0_*).

There is therefore a very high level of parallelization possible: each sharp problem can be solved using the FETI method described in Section 6.. In addition all equations except for the first are independent of each other, so after *u*_0_ is found the *u_i_* can all be computed independently And since the coefficient matrix is the same in all equations, most global objects, preconditioners, and decompositions can be computed only once and then reused across equations, reducing computational time.

For small uncertainties, the nonlinear part described by the last equation can be ignored. The results thus obtained for the displacements allow us to easily calculate the total range by using the expression *u*_0_ + Σ |*u_i_*| where the absolute value is considered component by component. However, for subsequent computations, such as stress and strain evaluation, it is better to keep the displacements in their affine form, as the information on the correlation between components of the stress tensor greatly improves the bounds of the von Mises equivalent stress.

**Figure 15.**
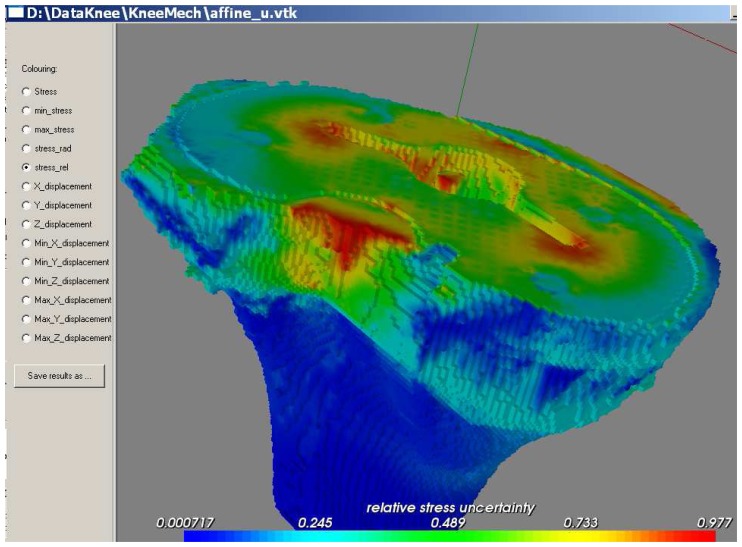
False-colour visualisation of the relative stress uncertainty after a simulation.

The program can calculate and display minimum, maximum, and average estimated stress values, and the relative uncertainty for each voxel (Figure 15). The most significant values that can be derived from uncertainty evaluation are maximum stress, and relative uncertainty. The latter is defined as the ratio between the range and the central value, for each noise symbol: for example, for the displacement we would have $\left|u_{i}^{\left(i\right)}/u_{0}^{\left(j\right)}\right|$ as the relative uncertainty of the *j*-component of the displacement, with respect to *ε_i_*. Adding up the relative uncertainty for each noise symbol gives the total relative uncertainty.

Areas where maximum stress is reached are critical from the mechanical point of view, as they allow the doctor to identify potential fractures that might result from the given prosthesis positioning. On the other hand, the relative uncertainties describe how sensible each area is to the uncertainty of the problem: their evaluation allows therefore a quick sensitivity analysis of the problem.

## 7. Discussion

The interdisciplinary research work described in the previous sections has come together in implementation into KneeMech [20], a software equipped with a friendly graphical user interface (GUI) for preoperative planning.

**Figure 16.**
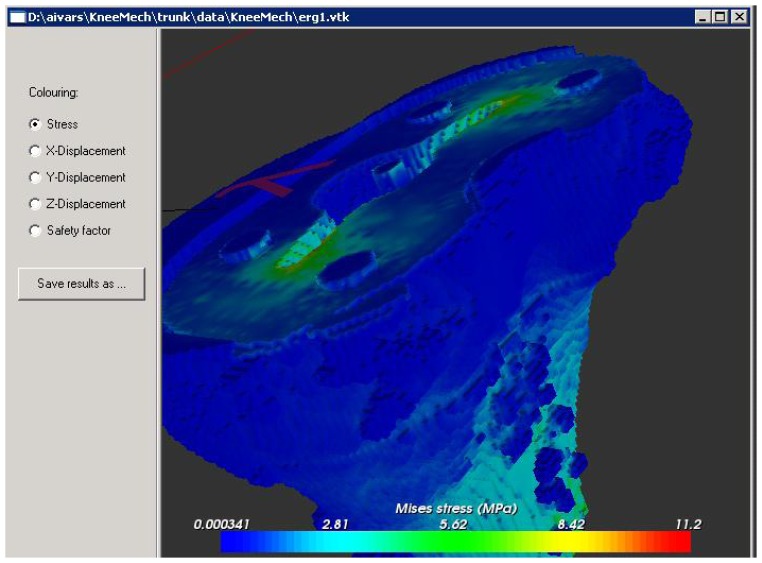
Sample screenshot from the KneeMech® program, showing the result of the simulation for a tibia.

The typical workflow starts with the selection of a CT scan dataset to use, which is automatically classified in a few seconds (Section 3.2.). The surgeon can then choose a cutting plane, a prosthesis model to use, and where to position it by manipulating the bone and prosthesis systems in a 3D environment. When she is satisfied with her pre-operative plan, she can launch the simulation. The system then takes care of the finite element discretisation (Section 5.) and the numerical simulation (Section 6.1.). The user can select whether sensitivity analysis must be used (Section 6.3.) or not (Section 6.1.). A *safety factor* can be also calculated, which measures the ratio between the computed equivalent stress and the critical stress. The result of the simulation is presented to the user in false colour (Figure 16). If the results are not satisfactory, the surgeon can then choose a different cutting plane, prosthesis, or position, and restart the simulation. Tibia and femur bones are handled separately.

Numerical examples have been ran to verify the effectiveness of the system. Real world applicability is being assessed by LIMA [2] with an extensive testing phase of the prototype implementation on real clinical cases. In this paper, we present a minimal set of numerical examples that show the influence of different parameters on the final results. In particular, we show how maximal stress and displacement in the bone are affected by varying the following parameters: bone morphology (structure), prosthesis size and position, bone stiffness, and loading direction.

**Figure 17.**
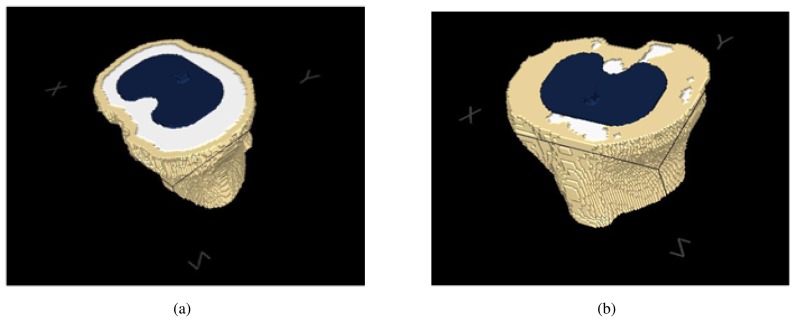
Support of prosthesis at cortical or spongy bone.

Bone structure is an extremely significant parameter; in Figure 17 we show a prosthesis touching mostly spongy bone (Figure 18(a)) and the same prosthesis placed on cortical bone (Figure 18(a)): the maximum equivalent von Mises stress in the first case is **5.5** MPa, while in the second case it's only **1.8** MPa. As expected, the contact of the prosthesis with spongy bone results in much higher stresses. It should be remarked that both examples are artificial and were chosen to pick up extreme cases: they are irrelevant for clinical practice, but show the importance of choosing an appropriate cutting plane and prosthesis size.

The influence of the prosthesis position is demonstrated by Table 4. Table 5 shows how the maximal equivalent stress and vertical displacement depend on the stiffness of the cortical bone. Finally, we loaded the same bone-prosthesis combination by 2 kN under different angles. The results are presented in Table 6 in terms of stress and displacement for angles 0° and 45°. Once again, prosthesis positioning has the most significant impact, showing the importance of preoperative planning.

Our system provides a significant advance over previous solutions. Its major novelty consists in providing most practical solutions to problems arising in such an interdisciplinary work. Other methods in the literature do not address most of the problems related to robust bone and prosthesis modelling from CT and mechanical data, as well as error handling. The latter is another important point of our system. Little work has been published addressing the way classification errors, data acquisition errors, and model uncertainties should be treated. Our system provides a simple solution with a solid theoretical background.

The only attempt we know about to build a complete system is *MedEdit* [1]. A quantitative comparison with this system is not possible since the authors do not provide timings or other quantitative analyses. Hence, we limit to a qualitative comparison. Our system has at least four advantages with respect to *MedEdit*. First, an error-bounded classification mechanism is provided instead of a simple region growing segmentation algorithm. User intervention in the classification stage is considerably reduced with respect to the *MedEdit* system. A single click on one slice suffices, while the segmentation algorithm in *MedEdit* requires some manipulations at the pixel level. Second, they extract a surface triangle model of the bones rather than a full solid tetrahedral mesh, as we do. This is a significant limitation for stress simulation. They solve this problem by allowing a mixed model, given by the surface mesh plus the voxel model. Voxels are decimated by a sub-sampling algorithm. In our system we decimate tetrahedra using a wiser strategy. Third, no means of estimating mechanical parameters is provided. They simply assume that parameters are available, which is not obviously true. Finally, *MedEdit* does not allow the introduction of uncertainties in the simulation. Our system can handle uncertainties at various steps of the workflow, such as tissue classification, parameter estimation, and finite element simulation.

The model used to solve the problem with uncertainties also introduces significant advantages over existing, interval-based uncertain FEM methods, by allowing fast, parallelisable methods to obtain first-order solutions and considerably reducing the overestimation of the result uncertainty.

The current system limitations are due to insufficient training data and computational limits. On the one hand, they increase the uncertainties of the problem, and on the other hand limit the order at which uncertainties can be handled, given the time limitations for the simulation in the clinical practice.

Higher order uncertainty handling comes at a considerable computation cost (as the increase is geometrical), but would ensure correctness of the results with larger uncertainties. Uncertainties can be reduced by correlating the mechanical properties of the bone with the patient's characteristics. This could be achieved by collecting the information in a large statistical database (Figure 1), which would include anonymous information on the patients' age, gender, physical characteristics such as height and weight, and presence of medical conditions such as osteoporosis or arthritis. This database would then be used by matching this information with the specific patient being treated, using the correlation with age, gender, height, and so on, to adapt the probability density functions used in the classification (Section 3.2.), or the maximum range of the bone mechanical parameters (Section 3.3.).

## Figures and Tables

**Figure 5.**
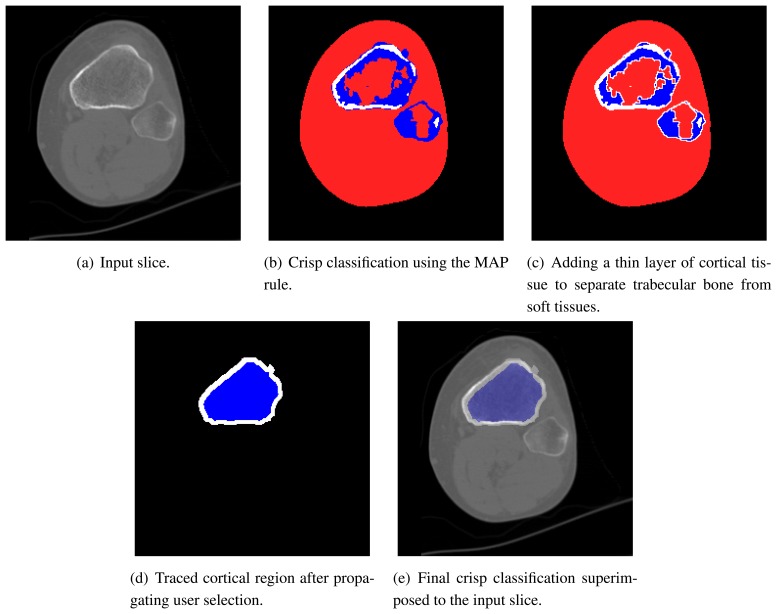
An example of a slice classified using our classification method. The colour codes are as follows: White for cortical bone, blue for trabecular bone, red for soft tissue, and black for background.

**Figure 7.**
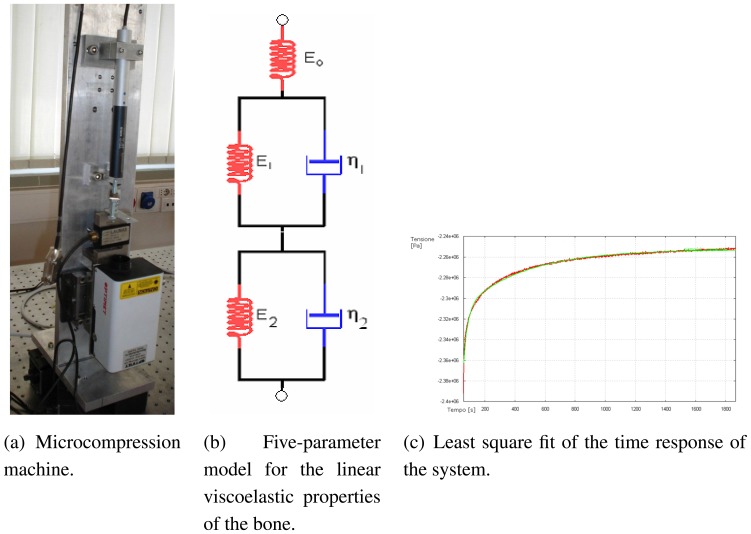
Determination of the mechanical viscoelastic parameters of the bone.

**Figure 9.**
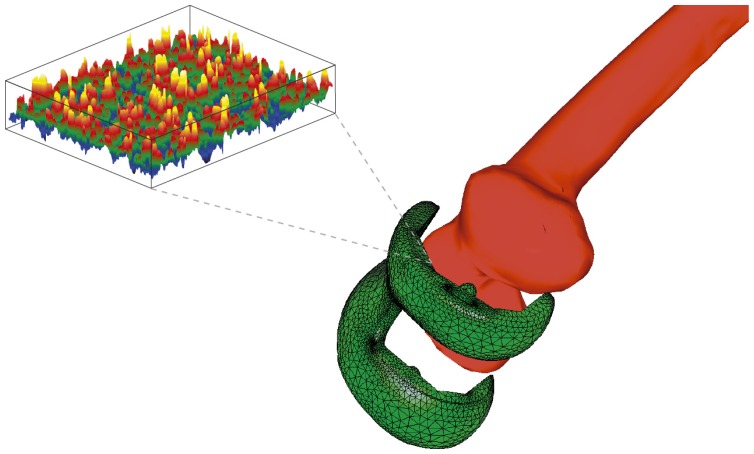
Bone-prosthesis-coating

**Table 1.** Parameter settings used for the acquisition of the knee CT data employed in our experiments.

Parameter	Value

Exposure	200 Sv
kVp	140 kiloVolt
Slice thickness	1.3 mm
Slice spacing	0.6 mm
Slice resolution	512 × 512 pixels
Number of slices	70–140

**Table 2.** Normal contact stiffness and friction coefficients for effective non-penetration condition in case of full microscopic contact. Parameters for *k_nn_*(*u*) using the expression (7) in case of solution-dependent micro-contact surface.

Coating	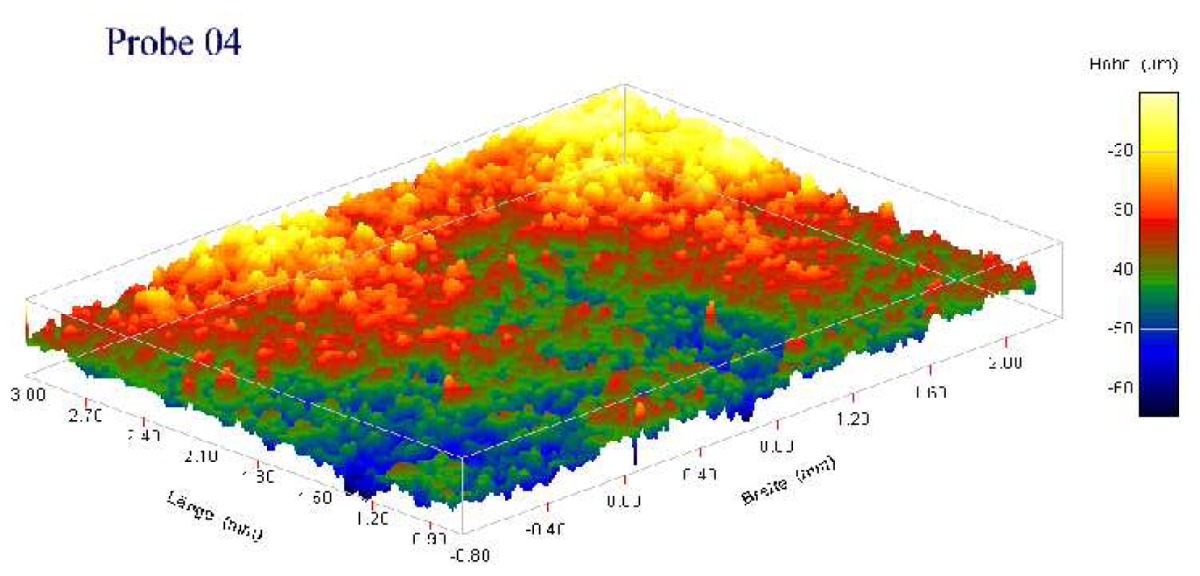	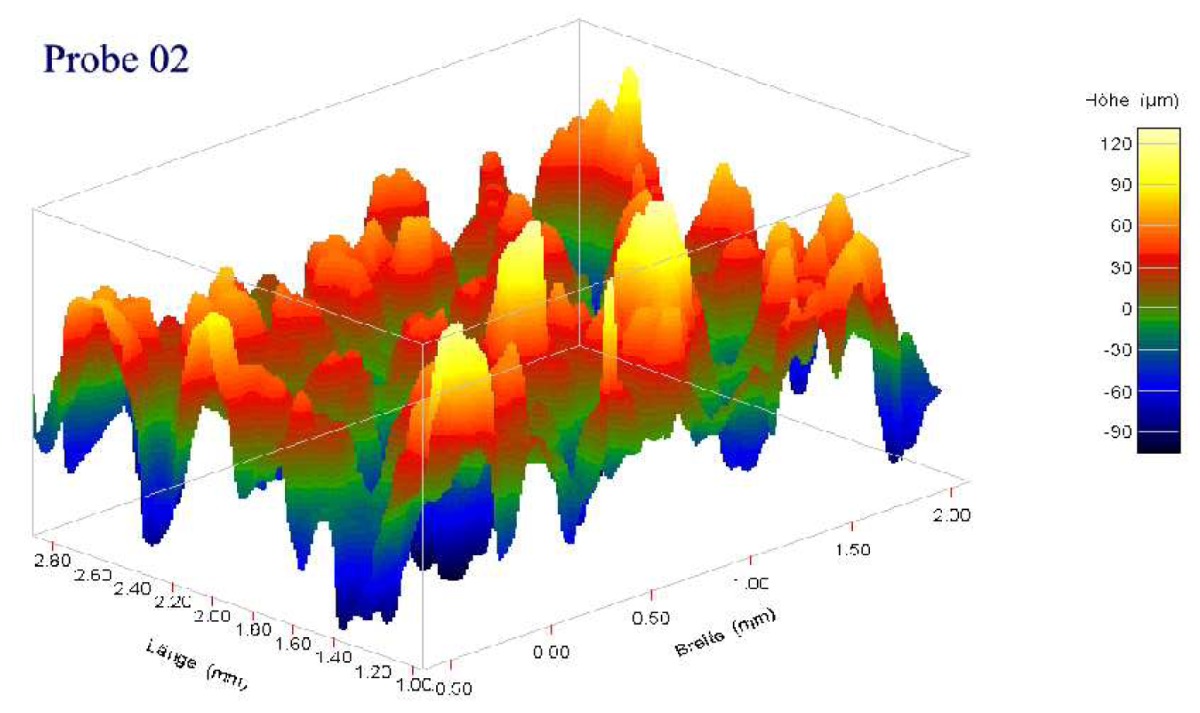	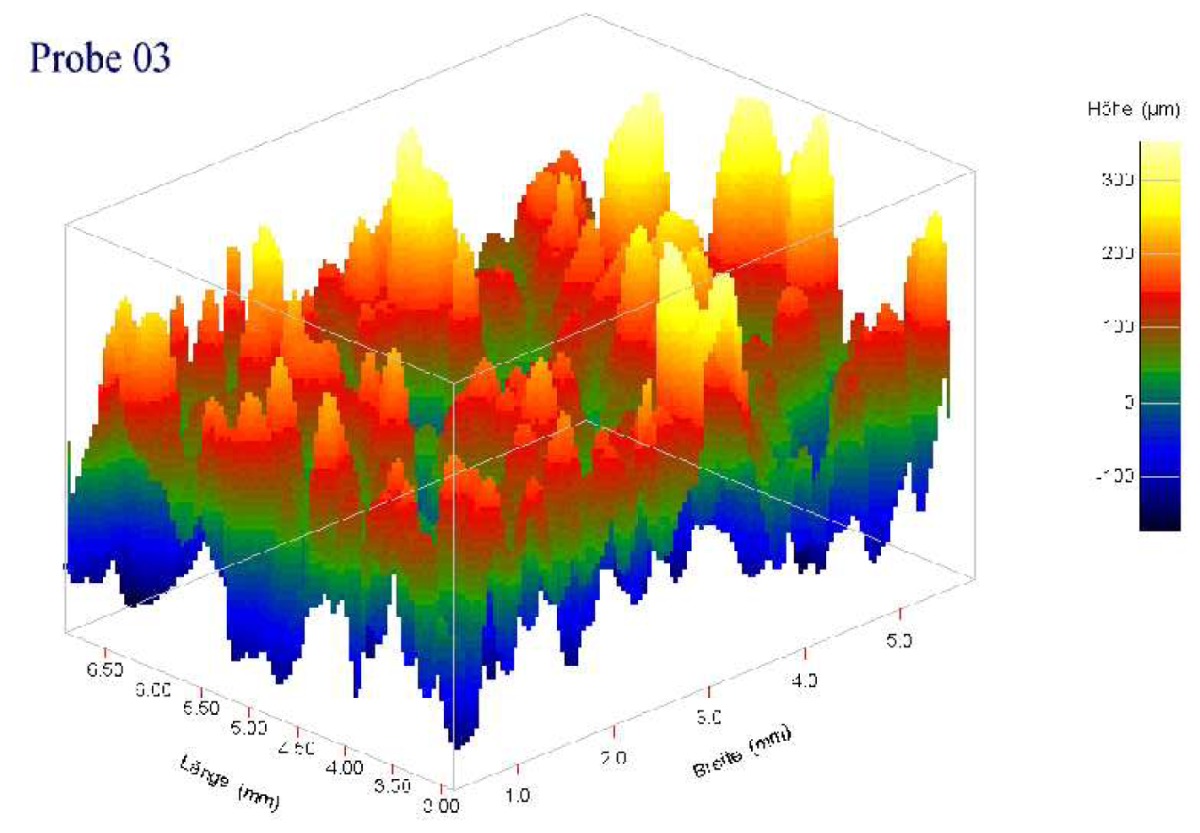
Roughness, Rt, *µm*	150 ± 50	200 ± 100	200 ± 100
Porosity, %	15 ± 10	30 ± 10	15 ± 10
Normal contact stiffness *k_nn_*	0.99	0.96	0.87
Friction coefficient	9.78e-04	5.45e-02	2.21e-01
Contact param. for *k_nn_*(*u*) from (7)			
*a_n_*	4.933230	2.337140	1.766300
*b_n_*	0.497877	0.382857	0.427986

**Table 3.** Number of nodes and elements for fine and coarse tetrahedral meshes of a tibia bone.

Coarsening Levels	Number of Nodes	Number of Elements	Node reduction	Time (sec.)
0	346 783	1 636 175	1.0	135
1	81 816	341 152	4.2	65
2	58 640	230 642	6.2	56
3	57 186	223 763	6.4	56

**Table 4.** Maximal equivalent stress via position of the prosthesis.

Position of the prosthesis	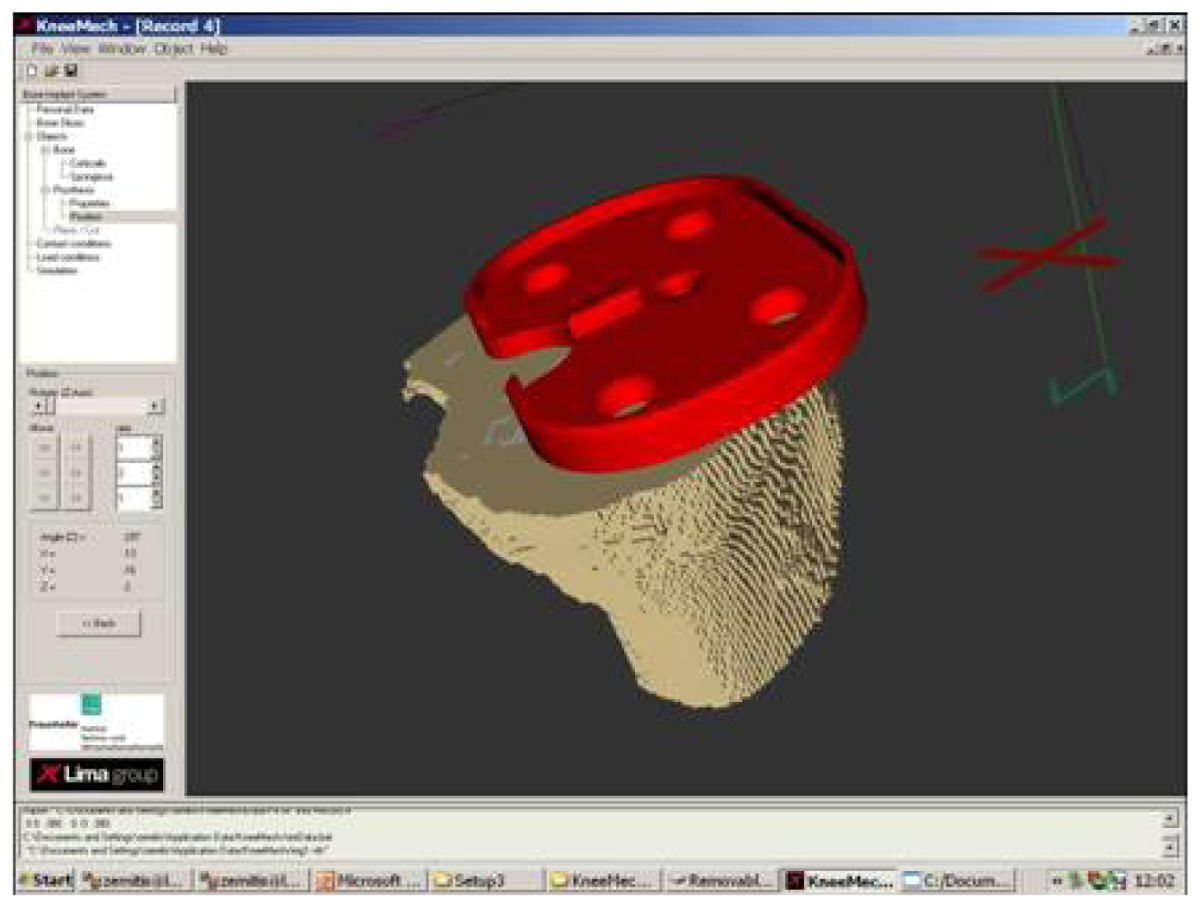	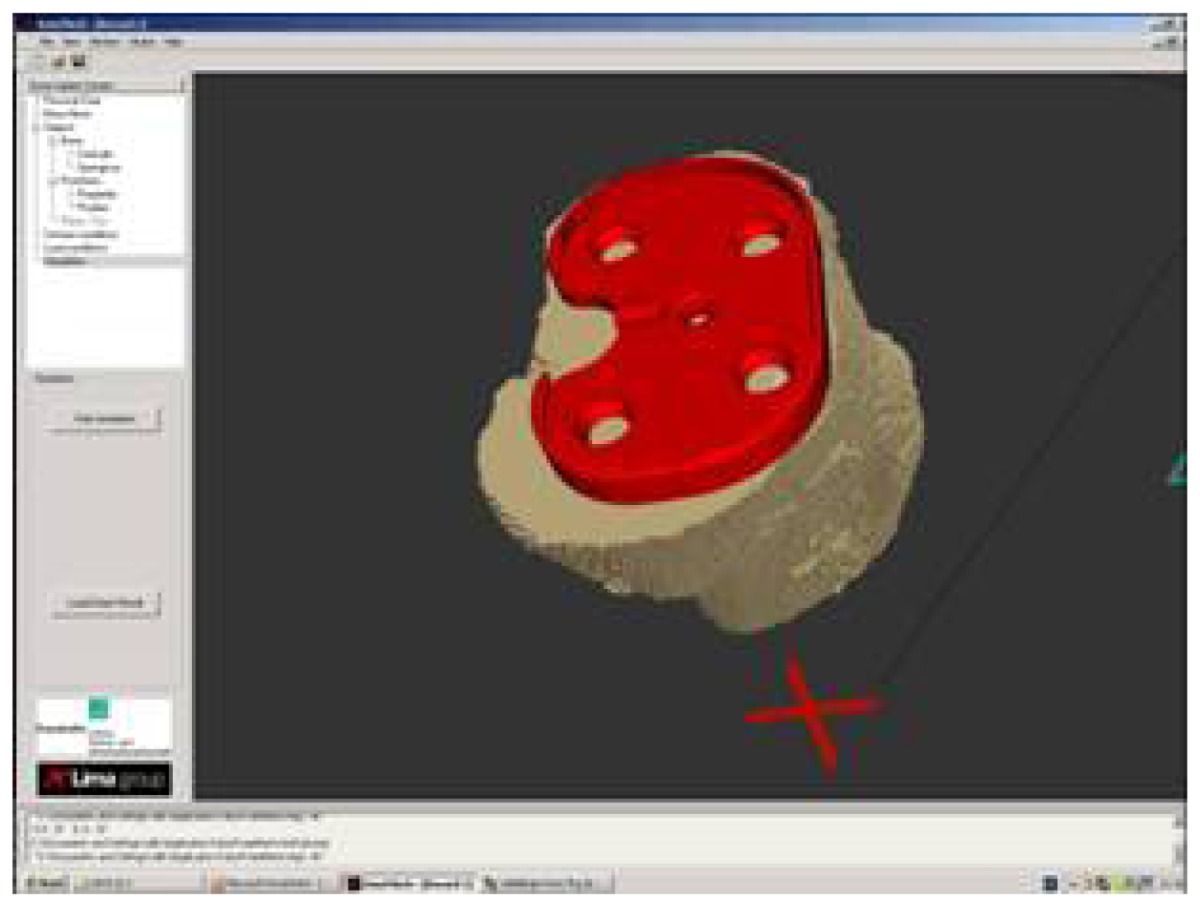	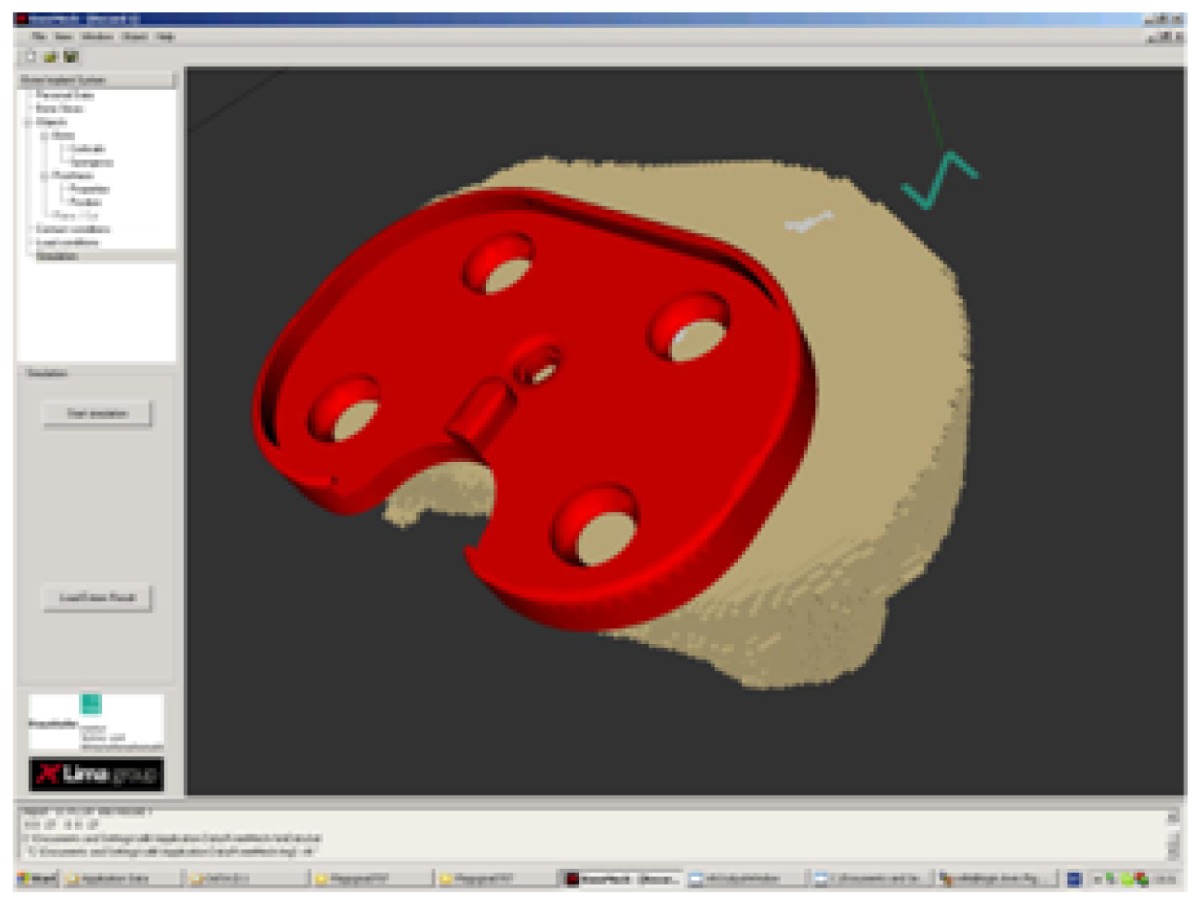
Equivalent stress (MPa)	1.25	1.1	1.18

**Table 5.** Maximal equivalent stress and vertical displacement via Young's modulus of cortical bone.

E (GPa)	Equivalent stress (MPa)	Vertical displacement (mm)
5	1.18	0.0316
10	1.19	0.0233
14.7	1.20	0.0152

**Table 6.** Stress and displacement for loading 2 kN under angles 0° and 45°

Angle, °	Equivalent stress (MPa)	Horizontal displacement (mm)
0	1.1	0.0152
45	1.2	0.00722

## Footnotes

∗ Data is usually acquired using spiral scanning. Pixel values are interpolated from spiral data

† For this reason, hereafter we will use the terms pixel and voxel interchangeably.
